# Artificial Intelligence in Planning for Spine Surgery

**DOI:** 10.1007/s12178-025-09992-5

**Published:** 2025-08-26

**Authors:** Iyad S. Ali, Yianni Bakaes, James S. MacLeod, Tony Y. Lee, Sia Cho, Wellington K. Hsu

**Affiliations:** 1 Department of Orthopaedic Surgery, Northwestern University Feinberg School of Medicine, 259 E Erie St, Chicago, IL 60611 USA; 2 Department of Orthopaedic Surgery & Rehabilitation, Loyola University Medical Center, Maywood, IL 60153 USA; 3 Department of Orthopaedic Surgery, Medical College of Wisconsin, Milwaukee, WI 53226 USA

**Keywords:** Artificial intelligence, Machine learning, Spine surgery, Surgical planning, Navigation, Real-time decision support

## Abstract

**Purpose of Review:**

There has been an expanding role of artificial intelligence (AI) and machine learning (ML) in spine surgery, particularly in operative planning, intraoperative navigation, and postoperative management. With a focus on patient-specific surgical strategies, AI technologies offer new possibilities for improving surgical accuracy, reducing risks, and enhancing patient outcomes in spine care.

**Recent Findings:**

AI models have shown strong accuracy in preoperative planning, with neural networks outperforming traditional algorithms in patient selection and outcome prediction. Advances in 3D modeling, supported by machine learning, enable efficient, patient-specific anatomical reconstructions, reducing manual segmentation time from hours to seconds. In intraoperative navigation, AI-driven virtual and augmented reality systems enhance screw placement precision and reduce radiation exposure by up to 90%, improving workflow and safety. Additionally, real-time AI-based decision support has decreased operative time and postoperative risks, while postoperative AI applications now support mortality risk stratification and discharge planning, yielding significant predictive accuracy for adverse events and extended stays.

**Summary:**

AI technologies are transforming spine surgery by increasing surgical precision, optimizing clinical workflows, and personalizing patient care. While challenges remain regarding data diversity and ethical considerations, ongoing innovations indicate that AI will continue to refine spine surgery through personalized and efficient care solutions.

## Introduction

Artificial intelligence (AI) refers to the use of computers to perform tasks that typically require objective reasoning and understanding [1]. A key subset of AI is machine learning (ML), which employs statistical and mathematical models to automatically identify patterns and enhance predictions of a target outcome without explicit programming [1]. Within ML, artificial neural networks (NN) are a set of algorithms that mimic the structure and function of the human brain [1]. Deep learning, a subfield of ML, utilizes artificial NNs to process large volumes of complex data, making it particularly useful in clinical applications [2]. Large language models (LLMs), a form of deep learning, generate human-like text by predicting sequential words based on statistical patterns learned from vast text data. These models are foundational to natural language processing (NLP) which have significant implications for clinical tasks [3].

As AI continues to evolve, there is growing emphasis on transparency and interpretability in clinical contexts. Explainable artificial intelligence (XAI) provides the core algorithmic result or prediction to the user along with an explanation that conveys insights into the confidence of the core prediction [2]. Additionally, generative adversarial networks (GANs) use two competing AI models: a generator that synthesizes artificial data and a discriminator that evaluates its authenticity. Through this process, GANs produce high-quality synthetic data which can be useful in medical imaging, data augmentation, or situations where datasets are limited [4].

In recent years, AI and ML have transformed fields across healthcare, especially in surgical specialties. In orthopaedics, AI’s role is rapidly expanding, offering innovative ways to optimize complex procedures and enhance patient care. For spine surgery, where precision and strategic planning are emphasized, AI has the potential to improve outcomes through data-driven, patient-specific surgical planning. This review provides an overview of current applications in spine surgery, examines their impact on operative planning, and discusses the implications for postoperative outcomes.

## Preoperative Modeling and Simulation

**Fig. 1:**
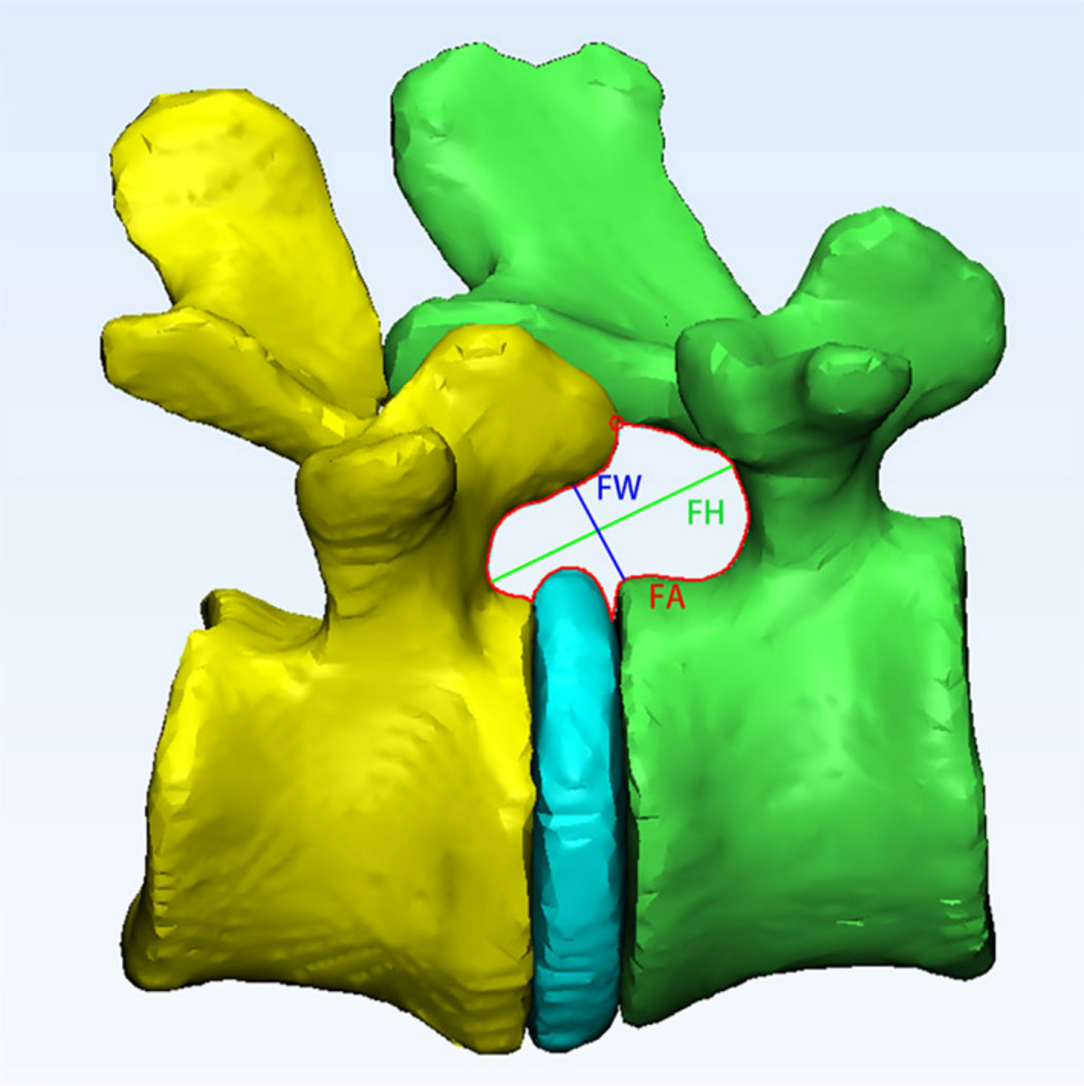
LIVF 3D model dimensions measured on lateral view. The LIVF height (FH) was defined as the longest distance between the cranio-caudal boundary (green line); the width (FW) was defined as the shortest distance between the postero‐inferior corner of the proximal vertebrae and the opposing boundary (blue line); and the area (FA) was drawn with the temporary boundaries set at 0.5 mm increments (red circle) according to the 3D LIVF model outline (red line). Reproduced from Chen et al. under Creative Commons Attribution-NonCommercial-NoDerivs License BY 4.0 [7]

AI modeling has demonstrated the capacity to improve preoperative planning by optimizing surgical decision making and increasing preparedness for planned procedures. Recent studies underscore the accuracy and potential of AI in spine surgery for creating customizable models and simulation of the planned surgery. Beyond the operative technique itself, both AI and ML can be used to facilitate preoperative planning and analyze surgical indications to ensure that the planned procedure is the most suitable for each individual patient. Tragaris et al. reviewed 46 studies implementing AI/ML, noting an overall mean accuracy of 74.9% in ML models for guiding clinical decision-making and surgical planning, with particular strengths in preoperative patient selection, anticipated cost, length of stay, and outcomes prediction [5]. Buchlak et al. compared NNs with traditional logistic regression algorithms, finding that these measures excelled in both accuracy and specificity, suggesting that an ML-facilitated learning algorithm may provide superior surgical planning [6]. After the decision on the optimal surgery is chosen for the individual patient, AI can then help further increase surgeon readiness through creation of custom models and simulation of the planned surgery. Three-dimensional modeling and simulation can be useful for precision in spine surgery, and AI has enabled remarkable advancements in this area. Machine learning enables the creation of patient-specific anatomical models, facilitating customized surgical approaches. For example, Chen et al. employed a deep learning model, 3D-UNet, to reconstruct lumbar intervertebral foramina (LIVF) in a shorter time compared to manual methods, while maintaining high accuracy [7]. They found that automated MRI segmentation based on deep learning algorithms led to no significant differences observed in foraminal area, height, and width of the 3D LIVF images when compared to manual images (Fig. 1). Notably, it took approximately 2.5 s to achieve each automated segmentation compared to 240 min for manual segmentation, demonstrating a substantial efficiency benefit when using AI [7].

**Fig. 2:**
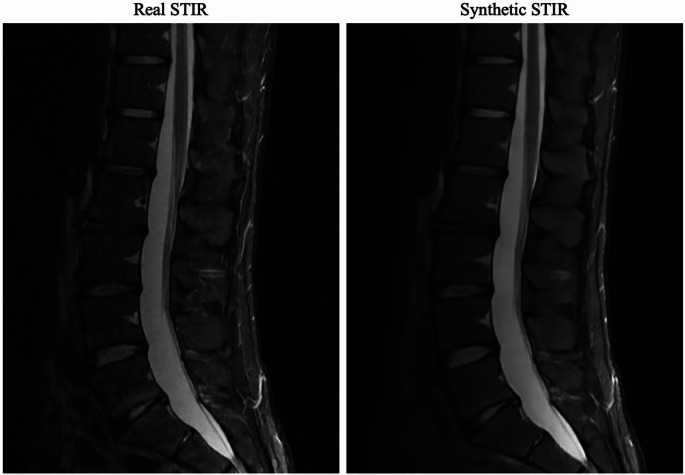
Randomly selected acquired and synthetic slice from a patient in the test set with evaluated high quality. Left: Slice from the acquired STIR volume. Right: Same slice from the synthetic STIR volume. Reproduced from Santilli et al. with permission from American Society of Neuroradiology [9]

Fan et al. used deep learning for semantic segmentation to assess surgical difficulty in percutaneous endoscopic transforaminal discectomy (PETD), achieving strong test-retest reliability, measured by intraclass correlation coefficients (ICC) between 0.947 and 0.971, and interobserver reliability of multiple measurements (ICC between 0.866 and 0.961) [8]. Moreover, AI-driven segmentation algorithms are consistently achieving over 90% accuracy in delineating vertebral structures from CT scans, supporting the creation of precise 3D reconstructions that can guide surgical planning [7, 8]. There has been a recent emergence of GAN-based modeling approaches for anatomic reconstruction in spine surgery. Santilli et al. developed an open-source GAN to create synthetic lumbar spine MRI STIR volumes from T1 and T2 sequences [9]. Radiologists evaluated synthetic volumes, demonstrating that these were of equal or better quality in 77% of test patients and had equal or decreased motion artifacts in 78% of patients (Fig. 2). This work highlights the utility of AI as a means for expediting imaging protocols in preoperative planning for spine surgery [9].

These findings suggest that AI utilization preoperatively can ensure surgical success by allowing surgeons to choose the best surgeries for each patient and allow the opportunity to practice the proposed surgery on an individualized model of the patient, all in a fraction of the time traditional methods would take. Furthermore, AI extends beyond anatomic modeling. Joshi et al. demonstrated that ML has given surgeons the ability to leverage far more accurate and individualized predictive tools to better inform patients [10]. Mani et al. integrated natural language processing (NLP) into traditional ML algorithms of tabular electronic health records, allowing for integration of free-text inputs to improve the personalized prediction of perioperative safety indicators in spinal surgery [11]. The multimodal NLP model exhibited superior performance in all outcome measures when compared to the baseline tabular model [11]. This exemplifies that AI can help surgeons tailor their clinical practice to address patients’ individual needs and create an opportunity for personalized medicine within spine surgery.

## Intraoperative Navigation and Real-time Decision Support

In the operating room, AI has further enabled enhancements in intraoperative navigation through virtual and augmented reality, as well as by providing robotic assistance. This novel technology has the potential to increase surgical precision, while simultaneously reducing the risk of iatrogenic injury and making surgery more time efficient. Comstock et al. developed a machine-vision image-guided system (MvIGS) for intraoperative navigation during pedicle screw placement in pediatric spine deformity correction surgery [12]. MvIGS uses advanced optics combining a light projector with 2 stereoscopic video cameras to create a 3D map of the patient’s anatomy and correlates this information with preoperative computerized tomography (CT). When compared to traditional 2D fluoroscopy-based navigation, MvIGS notably decreased intraoperative fluoroscopy time by 68% and radiation exposure by 66% while significantly reducing patient length of stay and operative time [12]. Similarly, Liebmann et al. integrated a deep neural network that segments the spine and simultaneously predicts its orientation into an augmented reality-based navigation system to develop a marker-less registration method for pedicle screw placement during lumbar spinal fusion. While sparing the time and radiation exposure required by traditional methods, this method demonstrated high clinical accuracy with a median screw trajectory error of 1.6° and an entry point error of 2.3 mm [13].

These advancements are not designed to replace the surgeon’s operative skills or decision-making ability but highlight the capacity of AI to deliver real-time supportive tools that can not only reduce surgical time, but also lower the intraoperative risks for the surgeon, operating room staff, and ultimately the patient. The intent of incorporating these tools is to perform safer, faster surgery, while maintaining high levels of precision. Bcharah et al. found that machine learning-enhanced navigation systems achieved accuracy rates between 96% and 99% when used for guiding pedicle screw insertions [14]. They also demonstrated that ML algorithms led to enhanced visualization, reduced radiation exposure (49 µSv with O-arm navigation vs. 556 µSv with fluoroscopy), increased efficiency, and potential for fewer intraoperative complications when compared with conventional approaches [14]. Ghaednia et al. highlighted the potential of AR and VR to improve navigation accuracy, while studies by Beyer and Abel demonstrated minimal deviations (0.95 mm and 0.8 mm, respectively) in screw placement with high accuracy during robotic-assisted cervical spine surgeries [15–17]. Additional studies by Laratta and Menta affirmed high clinical accuracy of screw placement (95.7% and 92%, respectively) in robotically guided spine procedures, and Devito reported a clinical acceptance rate exceeding 98% in these procedures [18–20]. With the assistance of these new techniques, procedural time and patient morbidity risk can both decrease by limiting repeat fluoroscopic imaging, trialing of different screw trajectories, and avoidance of damage to surrounding structures. Of note, Hu emphasized a learning curve, showing that success rates of robotic-assisted pedicle screw placement improve with increased experience, and that less screws are converted from robotic to manual placement with increasing experience [21]. This highlights that system familiarity and technique play a role in patient care with regard to virtual reality, and that more experience with devices during training may enhance outcomes [21]. As with any emerging technology in healthcare, widespread adoption will require time. However, the literature demonstrates that AI and ML can provide real-time, intraoperative improvements that will result in positive, long-term clinical outcomes [22].

## Prediction of Postoperative Outcomes

AI’s integration into postoperative care and outcome prediction has shown promising results, particularly in mortality risk stratification and complication forecasting. Karabacak et al. developed an interpretable ML model for predicting short-term postoperative outcomes including length of stay, non-home discharges, and readmissions following posterior cervical fusion, achieving an area under the receiver operating characteristic curve (AUROC) of up to 0.812 for non-home discharges [22]. The authors developed an open-access web application that allows clinicians to integrate ML predictions into clinical workflow, which has the potential to facilitate real-time decision making while optimizing clinical outcomes [22]. DiSilvestro et al., using a Naïve Bayes classifier on a cohort of 2,094 patients, achieved an AUC of 0.898 for predicting 30-day mortality, outperforming traditional models (AUC = 0.722) [23]. Gowd et al.’s logistic regression model demonstrated ML’s proficiency in predicting adverse events and transfusion requirements after anterior cervical discectomy and fusion (ACDF) [24]. Additional studies by Ogink and Cabrera explored ML’s predictive accuracy for discharge placements, with neural networks yielding c-statistics of 0.751 and 0.693, respectively [25, 26]. Shahrestani and Karhade et al. further highlighted the role of ML in predicting extended lengths of stay and nonroutine discharges, achieving AUCs up to 0.998, affirming ML’s utility in postoperative care planning [27, 28]. By having the power to anticipate clinical outcomes, surgeons have the opportunity to augment treatment plans to maximize patient satisfaction and minimize postoperative morbidity and mortality.

Given that each patient carries their own unique risk for postoperative adverse events, extended lengths of stay, and discharge somewhere other than home, ML’s ability to predict these factors may improve individual-based care. Mofatteh reviewed AI’s application across pre-, intra- and postoperative phases in spine surgery, suggesting that AI enhances diagnostic and prognostic outcomes [29]. For example, ML can be used for classification, regression and clustering to analyze large data sets, identify risk factors, and predict surgical complications and mortality among patients following a cervical discectomy and posterior lumbar spine fusion. Mofatteh also advocated for the use of ML in clustering patients, such as classifying the severity of lumbar disc degeneration on MRI or the pain progression of patients suffering from osteoporotic vertebral fracture, optimizing their management [29]. Li et al. developed an ensemble learning model, a form of ML, based on patient labs and demographics for predicting prolonged hospital stays after posterior spinal deformity surgery, providing empirical evidence of AI’s applicability in optimizing postoperative care plans [30]. Their published web-based calculator exemplifies the transition from theoretical models to practical tools that can aid clinicians in managing postoperative outcomes effectively [30]. The capacity to analyze large amounts of data in short periods of time allows ML models to predict negative outcomes, giving surgeons the opportunity to tailor surgical interventions to avoid negatives outcomes before they occur.

## Limitations

While the benefits of AI in spine surgery are substantial, limitations remain. Chen et al. emphasized the issue of dataset homogeneity, which restricts the generalizability of AI models [7]. Ethical considerations surrounding data privacy, patient consent, and transparency are also relevant, especially as AI plays a larger role in clinical decision-making. Additionally, transparent algorithms are still needed to ensure equitable access and trust in AI-enhanced surgical care. Finally, AI currently requires large datasets to offer predictive capabilities for postoperative management. Therefore, more transparent and validated AI models may still be warranted before clinicians can trust using AI predictions in their practice regarding postoperative patient care [31].

## Future Directions and Innovations

The future of AI in spine surgery holds significant promise, with ongoing advancements expected to further personalize surgical planning. Emerging research focuses on optimizing surgical techniques, predicting optimal surgical approaches, and recommending specific implants or devices based on patient comorbidities, pathologies, and variations in real time. However, the full realization of AI’s potential requires larger, diverse datasets to improve generalizability and model accuracy, particularly in the setting of postoperative management. Moreover, as AI continues to advance, it is poised to save time, reduce medical staff workload, and minimize healthcare costs by facilitating tailored treatment plans.

## Conclusion

Artificial intelligence and machine learning are transforming spine surgery, offering new possibilities in surgical planning, intraoperative navigation, and postoperative care. The studies discussed in this review underscore the potential of AI to enhance accuracy, reduce operative risks, and improve patient outcomes. While limitations exist, the ongoing development of AI tools and ethical frameworks suggests that machine learning will continue to shape the future of spine surgery in impactful ways.

## Key References

- Chen T, Su ZH, Liu Z, Wang M, Cui ZF, Zhao L, Yang LJ, Zhang WC, Liu X, Liu J, Tan SY, Li SL, Feng QJ, Pang SM, Lu H. Automated Magnetic Resonance Image Segmentation of Spinal Structures at the L4-5 Level with Deep Learning: 3D Reconstruction of Lumbar Intervertebral Foramen. Orthop Surg. 2022;14(9):2256-64. Epub 20220818. doi: 10.1111/os.13431. PubMed PMID: 35979964; PubMed Central PMCID: PMC9483078.

- This study demonstrates the feasibility of using a deep learning model for automated MRI segmentation of spinal structures at the L4-5 level significantly reducing the time and effort required for 3D reconstruction. It showcases how AI can improve surgical planning and preoperative visualization, key themes in our review.

- Bcharah G, Gupta N, Panico N, Winspear S, Bagley A, Turnow M, D’Amico R, Ukachukwu AK. Innovations in Spine Surgery: A Narrative Review of Current Integrative Technologies. World Neurosurg. 2024;184:127 − 36. Epub 20231228. doi: 10.1016/j.wneu.2023.12.124. PubMed PMID: 38159609.

- This review provides a comprehensize overview of AI-enhanced technologies currently being applied in spine surgery. It covers key advancements such as machine learning algorithms for outcome prediction, augmented reality navigation, and robotic assistance, aligning directly with the theme of our review on planning, navigation, and individualized surgical strategies.

- Karabacak M, Margetis K. Interpretable machine learning models to predict short-term postoperative outcomes following posterior cervical fusion. PLoS One. 2023;18(7):e0288939. Epub 20230721. doi: 10.1371/journal.pone.0288939. PubMed PMID: 37478157; PubMed Central PMCID: PMC10361477.

- This study demonstrates how interpretable machine learning models can be effectively used to predict short-term postoperative outcomes in spine surgery. Integrating predictive analytics into a web-based tool supports our review’s focus on outcome optimization and real-time clinical decision support.

## Data Availability

No datasets were generated or analysed during the current study.
